# PCP PRACTICES AND ATTITUDES AROUND COGNITIVE SCREENING IN RHODE ISLAND

**DOI:** 10.1093/geroni/igae098.2361

**Published:** 2024-12-31

**Authors:** Molly Lawrence, Matthew Howe, Alyssa De Vito, Charles Eaton, Louisa Thompson

**Affiliations:** Brown University, Providence, Rhode Island, United States; Brown University, Providence, Rhode Island, United States; Brown University, Providence, Rhode Island, United States; Brown University Medical School, Providence, Rhode Island, United States; Brown University Medical School, Lincoln, Rhode Island, United States

## Abstract

Primary care physicians (PCPs) are on the front lines for detecting cognitive changes in older adults but often have significant time constraints and are tasked with managing multiple conditions. For this reason, cognitive screening and follow up referrals need to be more efficient and effective. This study aims to evaluate how PCPs in Rhode Island (RI) manage memory concerns and cognitive screening. We created a 14-question, 5-minute survey which assessed several domains including level of familiarity, frequency of use and confidence with types of cognitive screening as well as barriers to screening and follow up. The survey was programed in Qualtrics and distributed through the RI Academy of Family Physicians. We received 48 responses and 26 of those were at least 50% complete. The MMSE (36.4%), MoCA (25.3%) and MiniCog (31.3%) were the top three screening assessments used. 77.3% of PCPs reported being “Very confident” or “Fairly confident” in conducting cognitive assessments, while only 2.3% were not at all confident. The three most frequently reported barriers to administering cognitive screening were: not have enough time for testing during appointments (23%), managing higher acuity conditions (13.2%) and not have enough time for counseling (13.2%). The top three barriers to referral to a memory specialist were: long wait times (31.5%), not enough resources (18.5%), and patient refusal (13.9%). Data collection is ongoing, but these preliminary findings provide insight into the current practices among RI PCPs and highlights time as a key limitation at multiples stages of memory screening and management by PCPs.

